# Clinical evaluation of a novel fixed-dose combination tablet (Sentorpil^®^ ForteGold) versus compounded powdered medications in dogs with myxomatous mitral valve disease: a randomized, double-blind study

**DOI:** 10.3389/fvets.2025.1622383

**Published:** 2025-08-12

**Authors:** Jiyoung Park, Jiyoon Lee, Sang-Joon Lee, Changbaig Hyun

**Affiliations:** Hyun Changbaig Animal Heart Institute, VIP Animal Medical Center, Seoul, Republic of Korea

**Keywords:** myxomatous mitral valve disease, fixed-dose combination tablet, canine cardiology, ForteGold, randomized clinical trial

## Abstract

**Introduction:**

Myxomatous mitral valve disease (MMVD) is a prevalent cardiac condition in older, small-breed dogs, often managed with multiple medications. Traditional administration involves compounded powdered mixtures, which may lead to dosing inaccuracies and reduced compliance.

**Methods:**

In a randomized, double-blind clinical trial, 60 client-owned dogs diagnosed with ACVIM stage C MMVD were assigned to receive either a novel fixed-dose combination tablet (Sentorpil^®^ ForteGold) or a compounded powdered mixture of torsemide, pimobendan, enalapril, and spironolactone. Treatments were administered twice daily over 56 days. Clinical signs, blood chemistry, electrolyte levels, thoracic radiography, echocardiography, and biomarkers (NT-proBNP, SDMA, and cPL) were evaluated at baseline and on days 7, 14, 28, and 56.

**Results:**

Both groups exhibited significant improvements in clinical signs, including exercise intolerance, appetite, respiratory effort, and coughing, with the ForteGold group showing earlier and more sustained responses. Blood chemistry and electrolyte levels remained within normal ranges, indicating a favorable safety profile. Radiographic assessments revealed a gradual decrease in vertebral heart score and normalization of lung fields by Day 56 in both groups. Echocardiographic parameters (LA/Ao, LVIDd/Ao, MVE) improved significantly, with the ForteGold group demonstrating earlier enhancements. NT-proBNP levels decreased in both groups, with a more pronounced reduction in the ForteGold group. No significant changes were observed in SDMA and cPL levels, suggesting stable renal and pancreatic functions.

**Discussion:**

The fixed-dose combination tablet (Sentorpil^®^ ForteGold) offers a clinically effective and safer alternative to compounded powdered medications for managing MMVD in dogs. Its formulation ensures accurate dosing, improved owner compliance, and enhanced clinical outcomes. Further studies with larger populations and extended follow-up periods are warranted to confirm these findings.

## 1 Introduction

Myxomatous mitral valve degeneration (MMVD) is the most prevalent acquired cardiac disease in older dogs, especially among small breeds (1). Characterized by thickening, elongation, and prolapse of the mitral valve leaflets, MMVD leads to mitral regurgitation, volume overload, and progressive cardiac remodeling. Common clinical signs include a heart murmur, cough, respiratory distress, and exercise intolerance. Without intervention, the disease often leads to congestive heart failure (CHF).

Medical management of MMVD focuses on alleviating symptoms and slowing disease progression using angiotensin-converting enzyme (ACE) inhibitors, positive inotropes such as pimobendan, and diuretics like torsemide (1–6). Although effective in palliating heart failure symptoms, these treatments do not reverse the underlying valvular degeneration. Long-term therapy often requires combination regimens and precise dosing, which can pose challenges in clinical practice.

In many Asian veterinary settings, cardiac medications are frequently compounded into powdered mixtures to simplify administration. However, this practice introduces variability in drug bioavailability, absorption, and dosing consistency. Inaccurate preparation and poor owner compliance can compromise therapeutic outcomes and increase the risk of side effects or treatment failure.

This study aimed to evaluate the clinical efficacy, safety profile, and practical advantages of (Sentorpil® ForteGold), a newly developed fixed-dose combination tablet, compared with traditional compounded powder formulations. The primary objective was to assess improvements in clinical symptoms, diagnostic imaging, and biomarkers, while monitoring adverse effects and dosing reliability.

## 2 Materials and methods

A prospective, randomized, double-blind clinical trial (NVRQS-1065) was conducted at VIP Animal Medical Center Chungdam, Seoul, from April 2022 to December 2023 (ethical approval obtained from the National Veterinary Research and Quarantine Service, Korea). Sixty client-owned dogs with echocardiographically confirmed MMVD at American College of Veterinary Internal Medicine (ACVIM) stage C were enrolled based on diagnostic guideline in elsewhere (1). Dogs with concurrent systemic illnesses were excluded. Investigators, owners, study monitors, and the statistician were blinded to treatment allocation. The randomization code was held by individuals uninvolved in the study. Neither investigators nor the study owner had access to the randomization list. Dogs that were randomized and received at least one dose of study medication comprised the intention-to-treat (ITT) population. Dogs in the ITT group who met all inclusion and no exclusion criteria formed the per-protocol (PP) population unless they reached the primary endpoint, were censored due to an event preventing continuation, or completed the study. Censoring occurred if a veterinarian prescribed chronic open-label cardiovascular medication for reasons unrelated to the primary endpoint. Subjects were randomly assigned to two treatment groups (*n* = 30 each). Group 1 received (Sentorpil® ForteGold) (Careside, Korea), a tablet containing torsemide (0.2 mg), pimobendan (0.6 mg), enalapril maleate (1.0 mg), and spironolactone (2.0 mg), dosed at one tablet per 2 kg of body weight, administered twice daily. The pill was specially formulated into a single tablet containing stabilizers and excipients to ensure that the four ingredients are well absorbed and remain stable. It was designed to be administered regardless of meals. Caregivers were instructed to give the pill either with a treat or as is, maintaining a 12-h interval between doses. Group 2 received a compounded powdered mixture consisting of torsemide (0.1 mg/kg Torem, Korea Menarini, Korea), pimobendan (0.3 mg/kg Vetmedin, Boehringer Ingelheim, Germany), enalapril (0.5 mg/kg Enapril, Ashish Life Science, Japan), and spironolactone (1 mg/kg Aldactone, Pfizer, Korea), administered twice daily. The compounded powdered mixture was also to be administered every 12 h regardless of meals, using the caregiver’s preferred method—either by dissolving it in water and administering it with a syringe, or by mixing it with a treat.

Clinical evaluation was done by method used elsewhere (2). Diagnostic imaging studies were performed by a single certified veterinarian (Hyun) using the same echocardiogram unit equipped with two sector cardiac probes of 2.4–8 MHz and 4–12 MHz (Vivid E95, GE Healthcare, Illinois, United States). Each patient underwent two-dimensional (2D), M-mode, color Doppler, spectral Doppler, and tissue Doppler analysis as per standard protocols (3, 7). All measurements were conducted or reviewed by a single author (Park).

Evaluations were conducted on Days 0, 7, 14, 28, and 56. Dogs exhibiting treatment failure, worsening clinical signs, or mortality from heart failure were excluded from further analysis. Assessment parameters included:

- Clinical evaluation: exercise intolerance (scored 1–4), appetite, respiratory effort, and coughing (Table 1).
- Serum biochemistry: blood urea nitrogen (BUN), creatinine, alanine aminotransferase (ALT), and alkaline phosphatase (ALP).
- Electrolytes: sodium, potassium, chloride, and phosphorus.
- Thoracic radiography: vertebral heart score (VHS), lung field infiltration grade (0–3). The severity of lung field infiltration was graded as follows: Grade 0 (None), Grade 1 (mild, primarily around the perihilar or central lung fields), Grade 2 (moderate, infiltrates extend from the perihilar region into the mid-lung fields), and Grade 3 (severe, widespread involvement, including perihilar, ventral, and caudal lung lobes).
- Echocardiography: left atrium-to-aorta ratio (LA/Ao), left ventricular internal diameter in diastole (LVIDd/Ao), and mitral valve E-wave velocity (MVE).
- Biomarkers: symmetric dimethylarginine (SDMA), N-terminal pro-brain natriuretic peptide (NT-proBNP), and canine pancreatic lipase (cPL).

**Table 1:** Scoring system used in clinical evaluation.

Variable	Score	Clinical correlate
Exercise tolerance	1 (Very good)	Dog moved around with ease and was able to fully exercise
	2 (Good)	Dog moved around with ease and was not able to fully exercise; ability to run was reduced
	3 (Moderate)	Dog was less active than normal, moved around a few times per day, avoided long walks
	4 (Poor)	Dog was inactive and would only get up to eat, drink, or urinate
Respiratory effort	1	Normal
	2	Mildly increased rate or effort
	3	Moderately labored
	4	Severe respiratory distress
Appetite	1	Increased
	2	Normal
	3	Decreased (2/3 normal)
	4	Markedly decreased (<2/3 normal)
Coughing	1	None
	2	Occasional (a few times a week)
	3	Frequent (a few times a day)
	4	Persistent (frequently during the day)

Statistical analyses were conducted using SPSS software (IBM, New York, United States). The minimum sample size required (*N* = 60) was determined based on a significance level of 0.05, a sample correlation coefficient (*r*) of 0.4 derived from a pilot study, and a desired power of 0.8. To account for potential loss of follow-up, a target sample size of 60 was established. The parameters (effect size = risk difference of 20%, *α* = 0.05, power = 80%) were used for sample size calculation. The primary endpoint was a composite of the development of left-sided CHF, or euthanasia for a cardiac reason, or death presumed to be cardiac in origin in dogs in the PP population. The normality of the data was assessed using the Kolmogorov–Smirnov normality test. Normally distributed data are presented as mean ± standard deviation while data not normally distributed are presented as median (interquartile range, IQR). For normally distributed variables, an unpaired *t*-test was employed to compare mean differences in variables between Group 1 (test) and 2 (control). In cases where variables were not normally distributed, the non-parametric Mann–Whitney *U*-test for independent samples was utilized to evaluate distributional differences between test groups. To assess within-group changes, the Wilcoxon signed-rank test was used for non-normally distributed (non-parametric) variables, and repeated measures ANOVA were applied for normally distributed (parametric) variables. We analyzed the longitudinal data using a linear mixed-effects model to account for repeated measures within subjects. The fixed effects included group (treatment vs. control) and time (baseline, Day 7, Day 14, Day 28, and Day 56) to assess differential changes over time between groups. We assumed an autoregressive (AR1) covariance structure to model the correlation between repeated measures over time. All statistical tests were two-sided on a 5% level of significance (*α* = 0.05) with Bonferroni correction for multiple analyses, in order not to inflate the type II error, using SPSS software (IBM, New York, United States).

## 3 Results

The study included dogs of various breeds. In the Group 2 (control group), the breeds were as follows: Maltese (15), Pomeranian (2), Chihuahua (3), Poodle (2), Japanese Spitz (1), Bichon Frisé (2), Cocker Spaniel (1), Golden Retriever (1), and Mixed breeds (2). In the Group 1 (test group), the breeds included: Maltese (16), Pomeranian (6), Chihuahua (1), Poodle (3), Shih Tzu (2), and Mixed breeds (2). Regarding sex distribution, the Group 2 consisted of 14 males and 16 females, while the Group 1 included 19 males and 11 females. The average body weight and age of dogs in the Group 2 were 4.76 ± 2.86 kg and 11.66 ± 2.45 years, respectively. In the Group 1, the average body weight was 4.10 ± 1.36 kg, and the average age was 12.96 ± 2.77 years. There were no statistically significant differences between the two groups in terms of breed composition, age, or body weight. Of the 60 enrolled dogs, 56 completed the study. Four dogs (two from each group) were censored due to progression of heart failure or treatment noncompliance. However, all 60 dogs were included in the final statistical analysis.

### 3.1 Clinical outcomes

**Figure 1:**
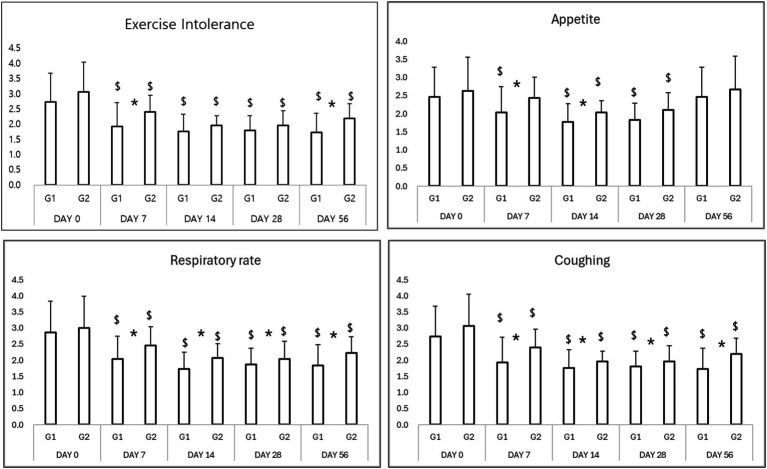
Results of clinical evaluation in study period. ^*^p < 0.05 Group 1 vs. Group 2 ^$^*p* < 0.05 compared to Day 0.

Both treatment groups showed significant improvement in clinical signs, including exercise intolerance, respiratory effort, appetite, and coughing scores (Table 2). By Day 7, 86% of dogs in the Group 1 (test group) showed marked clinical improvement versus 65% in the Group 2 (control group; *p* < 0.05). This trend persisted throughout the study period, with Group 1 maintaining a more consistent and sustained improvement (Figure 1).

**Table 2:** Results of clinical evaluation in study period.

Parameters		Day 0		Day 7		Day 14		Day 28		Day 56	
		G1	G2	G1	G2	G1	G2	G1	G2	G1	G2
Exercise intolerance	Median	2	2	2^*,$^	2	2	2.5	2	2	2^*,$^	2
	Range	2–3	2–4	2–3	1–2	2–2	1–2	2–3	2–2	2–2	2–2
Appetite	Median	2	2	2^*,$^	2	2^*,$^	2^$^	2^$^	2^$^	2	2
	Range	2–3	2–3	1–2	2–2	2–3	2–4	2–3	2–2	2–2	2–4
Respiratory effort	Median	2	2	2^*,$^	2^$^	2^*,$^	3^$^	2.5^*,$^	2^$^	2^*,$^	2^$^
	Range	2–4	2–3	1–2	2–2	1–2	2–4	2–3	2–2	2–2	2–3
Coughing	Median	2	2	2^*,$^	2^$^	2^*,$^	3.5^$^	2^*,$^	2^$^	2^*,$^	2^$^
	Range	2–4	1–3	1–2	2–2	1–2	2–4	2–3	2–2	2–2	2–2

^*^p < 0.05 Group 1 vs. Group 2 ^$^*p* < 0.05 compared to Day 0.

### 3.2 Serum chemistry and electrolytes

**Figure 2:**
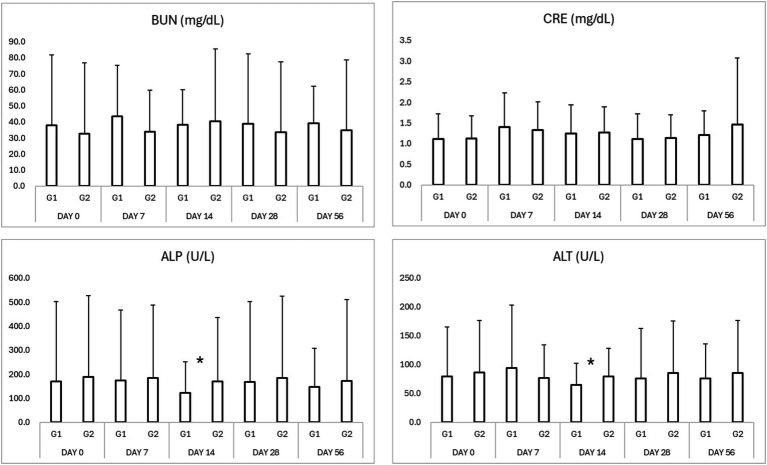
Results of serum biochemistry in study period. ^*^p < 0.05 Group 1 vs. Group 2 ^$^*p* < 0.05 compared to Day 0. BUN, blood urea nitrogen; CRE, creatinine; ALP, alkaline phosphatase; ALT, alanine aminotransferase.

**Figure 3:**
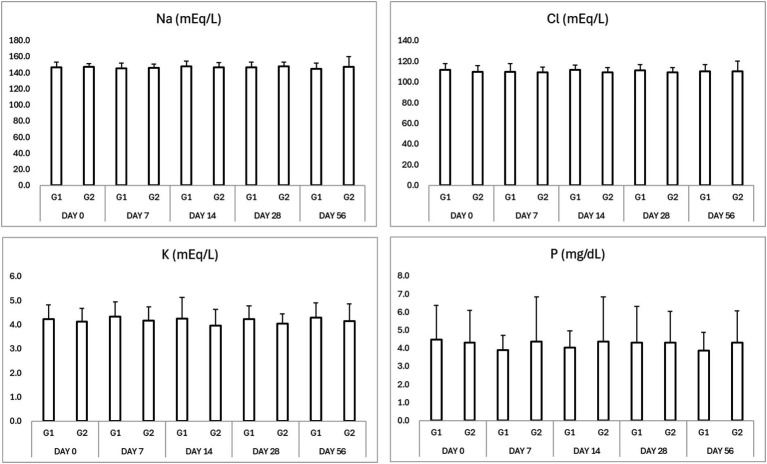
Results of serum electrolytes in study period. ^*^p < 0.05 Group 1 vs. Group 2 ^$^*p* < 0.05 compared to Day 0. Na (sodium), K (potassium), Cl (chloride), and P (phosphorus).

No significant adverse changes in liver enzymes (ALT, ALP), renal parameters (BUN, creatinine), or electrolytes (sodium, potassium, chloride, and phosphorus) were observed in either group (Tables 3, 4; Figures 2, 3). This indicates that both treatment regimens were well tolerated, with no evidence of hepatotoxicity, nephrotoxicity, or electrolyte imbalance.

**Table 3:** Summary of blood chemistry, electrolytes, biomarkers, thoracic radiography, and echocardiography in study period.

Parameters	Range	DAY 0		DAY 7		DAY 14		DAY 28		DAY 56	
		G1	G2	G1	G2	G1	G2	G1	G2	G1	G2
BUN (mg/dL)	16–36	38.1	32.6	43.6	33.9	38.1	40.5	38.9	33.4	39.1	34.9
	±	43.7	44.3	31.8	26.0	22.1	45.1	43.4	44.0	23.1	43.9
CRE (mg/dL)	0.8–1.6	1.1	1.1	1.4	1.3	1.3	1.3	1.1	1.1	1.2	1.5
	±	0.6	0.5	0.8	0.7	0.7	0.6	0.6	0.6	0.6	1.6
ALP (U/L)	14–111	169.0	189.3	173.3	184.5	122.0^*^	169.8	167.8	184.7	146.5	172.4
	±	333.8	338.7	292.8	302.7	129.2	266.4	334.1	339.8	160.2	337.4
ALT (U/L)	12–130	79.4	85.9	94.1	76.5	64.8^*^	79.3	76.4	85.5	75.6	85.3
	±	86.2	90.2	108.9	57.4	37.4	48.9	86.6	90.5	60.7	90.9
P (mg/dL)	3.1–7.5	4.5	4.3	3.9	4.4	4.0	4.4	4.3	4.3	3.9	4.3
	±	1.9	1.8	0.8	2.5	0.9	2.4	2.0	1.7	1.0	1.8
Na (mEq/L)	149–157	146.6	147.1	145.2	145.8	147.8	146.8	146.8	147.6	144.8	147.5
	±	6.3	4.0	6.6	4.9	6.3	5.5	6.5	5.3	6.9	12.7
K (mEq/L)	3.3–4.5	4.2	4.1	4.3	4.2	4.2	3.9	4.2	4.0	4.3	4.1
	±	0.6	0.5	0.6	0.6	0.9	0.7	0.5	0.4	0.6	0.7
Cl (mEq/L)	117–127	111.4	109.8	109.9	109.0	111.5	109.3	111.0	109.3	110.2	110.2
	±	6.3	5.7	7.8	5.0	4.9	4.2	6.0	4.7	6.6	9.8
cPL (ng/mL)	<50	109.7	91.6	89.7	75.4	101.5	62.9	112.7	82.2	114.5	89.3
	±	168.6	108.7	102.4	83.7	123.1	31.1	168.4	74.3	239.5	83.2
SDMA (μg/dL)	<17	15.5	17.3	17.6	17.1	18.0	16.8	16.1	17.4	19.6	17.3
	±	9.0	11.7	11.1	10.0	11.9	11.6	8.7	10.5	17.0	10.4
NT-proBNP (pmol/L)	<900	6,783	6,916	4533^*,$^	5,066^$^	2,783^$^	2,700^$^	2,433^$^	2,616^$^	2150^*,$^	2,683^$^
	±	1985	1916	1,425	1,512	1,393	1,494	1,179	1,406	1,018	1,476
VHS	8.5–10.5	12.3	11.4	11.8	11.1	11.5	10.9	11.7	11.0	11.2	11.2
	±	1.0	1.1	1.0	0.9	0.9	0.7	0.4	0.9	0.8	1.5
Lung field	0	0.8	0.7	0.3^*,$^	0.4	0.2^*,$^	0.3^$^	0.2^*,$^	0.3^$^	0.2^*,$^	0.3^$^
	±	0.9	0.8	0.5	0.7	0.4	0.5	0.4	0.7	0.4	0.7
LA/Ao	<1.4	2.3	2.3	2.0	2.0	2.0^$^	1.9	1.9^$^	2.0^$^	1.9^$^	2.1^$^
	±	0.3	0.3	0.4	0.4	0.5	0.4	0.3	0.4	0.4	0.5
LVIDd/Ao	<2.0	2.6	2.5	2.4^*,$^	2.2^$^	2.4^$^	2.2^$^	2.3^$^	2.2^$^	2.3^$^	2.3^$^
	±	0.5	0.3	0.5	0.2	0.5	0.2	0.3	0.2	0.5	0.4
MVE (m/s)	<0.8	1.2	1.3	1.0^*,$^	1.1^$^	0.9^*,$^	1.0^$^	0.9^*,$^	1.0^$^	0.9^*,$^	1.1^$^
	±	0.2	0.2	0.2	0.3	0.3	0.2	0.2	0.2	0.2	0.3

^*^p < 0.05 Group 1 vs. Group 2 ^$^*p* < 0.05 compared to Day 0.

**Table 4:** *P*-values and confidence intervals for change from baseline analysis of efficacy endpoints.

Parameters/ day		G1		G2			G1		G2	
		*p*-value	95%CI	*p*-value	95%CI		*p*-value	95%CI	*p*-value	95%CI
Exercise intolerance	7	<0.0001	−1.5 to −0.5	<0.0001	−1 to 0	VHS	0.24	0.1780 to 0.7486	0.401	0.01479 to 0.5985
	14	<0.0001	−1 to 0	<0.0001	−1.5 to −0.5		0.051	0.4331 to 1.1402	0.19	0.1768 to 0.7032
	28	<0.0001	−1 to 0	<0.0001	−1.5 to −0.5		0.39	0.2089 to 0.9911	0.75	0.1136 to 0.6731
	56	<0.0001	−1 to 0	<0.0001	−1 to 0		0.1542	0.6383 to 1.5683	0.6644	−0.4522 to 0.6989
Appetite	7	<0.0001	−1 to 0	<0.0001	−1 to 0	Lung field	0.0041	0.1607 to 0.7726	0.0029	0.09872 to 0.4346
	14	<0.0001	−1 to 0	<0.0001	−1 to 0		0.0014	0.2519 to 0.9481	0.0529	0.1297 to 0.6703
	28	<0.0001	−1 to 0	<0.0001	−1 to 0		0.0027	0.2253 to 0.9747	0.0137	0.08105 to 0.6523
	56	0.0663	0 to 1	0.061	0 to 1		0.0024	0.2174 to 0.9159	0.0137	0.08105 to 0.6523
Respiratory effort	7	<0.0001	−1.5 to −0.5	<0.0001	−1.5 to −0.5	LA/Ao	0.19	−0.03493 to 0.1683	0.1963	−0.02914 to 0.1358
	14	<0.0001	−1 to 0	<0.0001	−1.5 to −0.5		<0.0001	−0.4882 to −0.2398	0.0597	−0.003213 to 0.1499
	28	<0.0001	−1 to 0	<0.0001	−1.5 to −0.5		<0.0001	−0.4759 to −0.2921	0.0001	−0.4050 to −0.1503
	56	<0.0001	−1.5 to −0.5	<0.0001	−1 to 0		<0.0001	−0.5228 to −0.2252	<0.0001	−0.4050 to −0.1503
Coughing	7	<0.0001	−1.5 to −0.5	<0.0001	−1.5 to −0.5	LVIDd/Ao	0.0002	−0.2668 to −0.09252	<0.0001	−0.4010 to −0.2003
	14	<0.0001	−1 to 0	<0.0001	−1.5 to −0.5		0.0004	−0.2770 to −0.08896	<0.0001	−0.3953 to −0.1927
	28	<0.0001	−1.5 to −0.5	<0.0001	−1.5 to −0.5		<0.0001	−0.4268 to −0.1792	<0.0001	−0.3722 to −0.1691
	56	<0.0001	−1 to 0	<0.0001	−1 to 0		0.0007	−0.4099 to −0.1228	0.0042	−0.3492 to −0.07209
BUN	7	0.4157	−19.0646 to 8.0980	0.8271	−13.0205 to 10.4872	MVE	<0.0001	−0.3179 to −0.1921	<0.0001	−0.2592 to −0.1141
	14	0.9904	−10.2226 to 10.1026	0.3875	−26.2170 to 10.4770		<0.0001	−0.4004 to −0.2096	<0.0001	−0.3342 to −0.1658
	28	0.3452	−2.6095 to 0.9429	0.5914	−3.8304 to 2.2238		<0.0001	−0.3456 to −0.1911	<0.0001	−0.3110 to −0.1490
	56	0.8725	−13.4928 to 11.5128	0.1836	−5.8033 to 1.1633		<0.0001	−0.4004 to −0.2096	<0.0001	−0.3071 to −0.1262
CRE	7	0.2015	−0.05522 to 0.4352	0.2621	−0.9085 to 0.2551	cPL	0.5546	−81.643 to 121.643	0.3964	−54.6556 to 22.2756
	14	0.1123	−0.2998 to 0.03318	0.1402	−0.08825 to 0.1882		0.7884	−84.383 to 100.903	0.098	−63.0544 to 5.6344
	28	0.7868	−0.02164 to 0.02830	0.5803	−0.06210 to 0.03544		0.3559	−12.334 to 6.514	0.4459	−34.0127 to 15.3594
	56	0.4788	−0.3465 to 0.1665	0.2368	−0.9157 to 0.2357		0.9233	−154.728 to 145.141	0.8593	−27.8904 to 23.4037
ALP	7	0.8036	−38.7320 to 30.2654	0.7137	−21.6989 to 31.2989	SDMA	0.08393	−0.9105 to 5.5557	0.9071	−4.4106 to 3.9306
	14	0.3775	−60.2883 to 154.3550	0.3211	−19.9996 to 58.9863		0.1012	−0.4737 to 6.2866	0.7879	−4.6641 to 3.5707
	28	0.3521	−1.4337 to 3.9003	0.3869	−6.1105 to 15.3105		0.0805	−0.3216 to 3.4183	0.9578	−3.7361 to 3.9361
	56	0.5819	−60.3084 to 105.4418	0.2539	−12.8479 to 46.7946		0.1528	−0.7460 to 12.2944	0.9986	−3.7246 to 3.7313
ALT	7	0.5063	−59.3800 to 29.9800	0.3087	−9.2190 to 28.1523	NT-proBNP	<0.0001	−2768.3119 to −1731.6881	<0.0001	−2478.1855 to −1221.8145
	14	0.2365	−10.1049 to 39.3049	0.5667	−16.8616 to 30.1949		<0.0001	−4754.8169 to −3245.1831	<0.0001	−4919.8475 to −3513.4859
	28	0.2458	−2.1796 to 8.1796	0.9319	−9.0895 to 9.8895		<0.0001	−3757.2564	<0.0001	−4944.1512 to −3655.8488
	56	0.8139	−28.6689 to 36.2022	0.9026	−9.5967 to 10.8301		<0.0001	−5237.6909 to −4028.9757	<0.0001	−4825.2314 to −3641.4353
Na	7	0.1393	−0.4950 to 3.3616	0.0984	−0.2571 to 2.8571	Cl	0.1797	−0.7313 to 3.7313	0.5653	−1.2077 to 2.1677
	14	0.3068	−3.4603 to 1.1270	0.8082	−1.7148 to 2.1815		0.9591	−2.7051 to 2.5718	0.6555	−1.2381 to 1.9381
	28	0.2458	−0.5453 to 0.1453	0.5897	−2.3754 to 1.3754		0.2187	−0.2716 to 1.1383	0.4773	−0.9202 to 1.9202
	56	0.1561	0.7550 to 4.4883	0.8484	−5.2206 to 4.3206		0.2929	−1.1215 to 3.5882	0.8604	−4.4260 to 3.7194
K	7	0.2817	−0.2674 to 0.08069	0.5411	−0.2010 to 0.1077	P	0.0691	−0.04831 to 1.2083	0.8926	−1.0676 to 0.9343
	14	0.92	−0.2824 to 0.2557	0.1448	−0.06447 to 0.4178		0.0698	−0.03920 to 0.9459	0.8697	−1.0241 to 0.8708
	28	0.8797	−0.08264 to 0.09597	0.2077	−0.05088 to 0.2242		0.1925	−0.08347 to 0.3968	0.923	−0.2197 to 0.1997
	56	0.6825	−0.2776 to 0.1843	0.9755	−0.2234 to 0.2167		0.0555	0.01305 to 1.1936	0.9391	−0.3674 to 0.3407

### 3.3 Biomarker analysis

**Figure 4:**
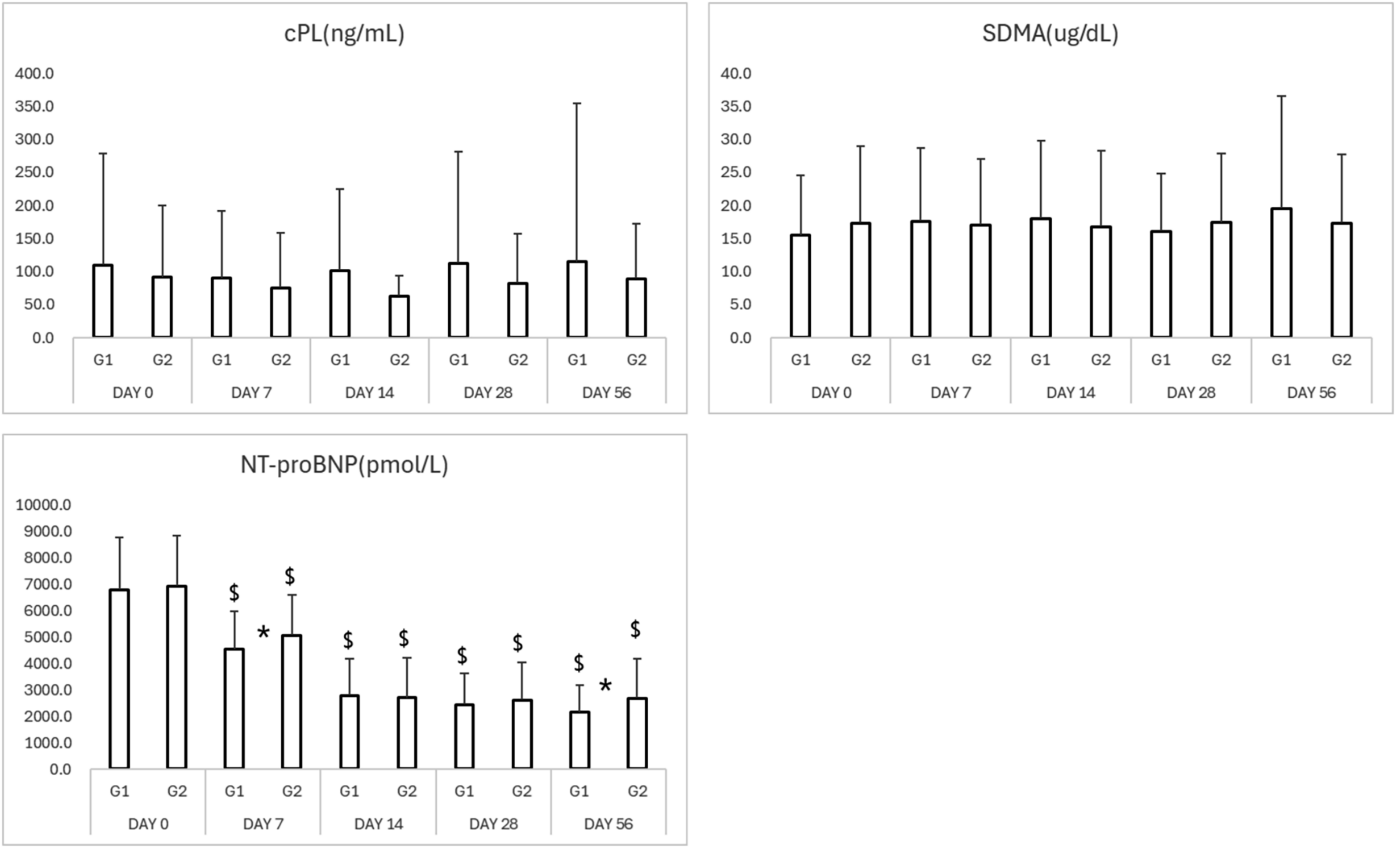
Results of biomarkers in study period. ^*^p < 0.05 Group 1 vs. Group 2 ^$^*p* < 0.05 compared to Day 0. cPL, canine pancreatic lipase; SDMA, symmetric dimethylarginine; NT-proBNP, N-terminal pro B-type natriuretic peptide.

NT-proBNP levels significantly decreased from baseline in both groups by Day 7 (Table 3; Figure 4). The test group exhibited a more rapid and consistent decline, maintaining lower NT-proBNP concentrations through Day 56 (*p* < 0.05). SDMA and cPL levels remained within normal limits in both groups, with no significant fluctuations, suggesting preserved renal and pancreatic function (Figure 4).

### 3.4 Radiographic findings

**Figure 5:**
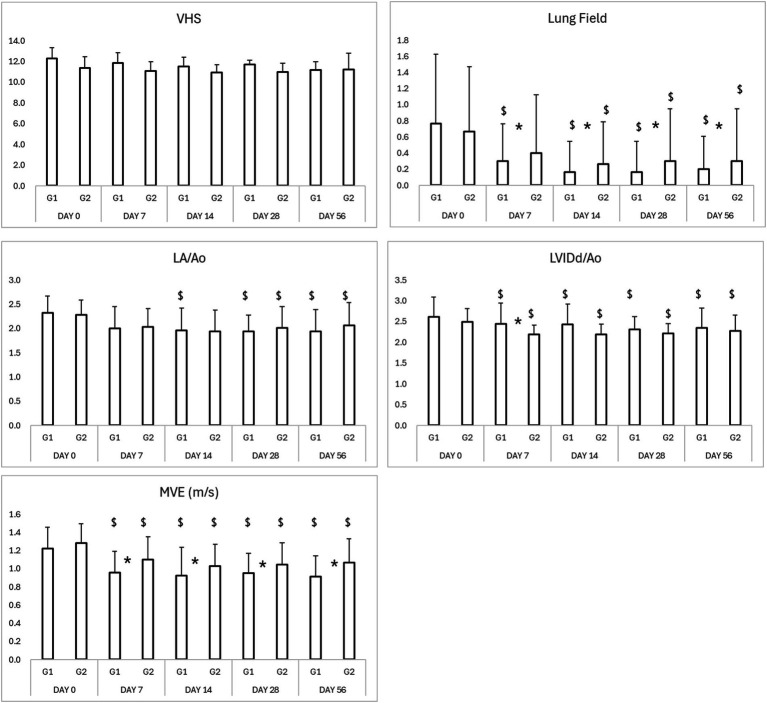
Results of thoracic radiography and echocardiography in study period. ^*^p < 0.05 Group 1 vs. Group 2 ^$^*p* < 0.05 compared to Day 0. VHS, vertebral heart score; LA/Ao, left atrium-to-aortic root ratio; LVIDd/Ao, left ventricular internal diameter in diastole-to-aortic root ratio; MVE, mitral valve E-wave, early diastolic filling velocity.

**Figure 6:**
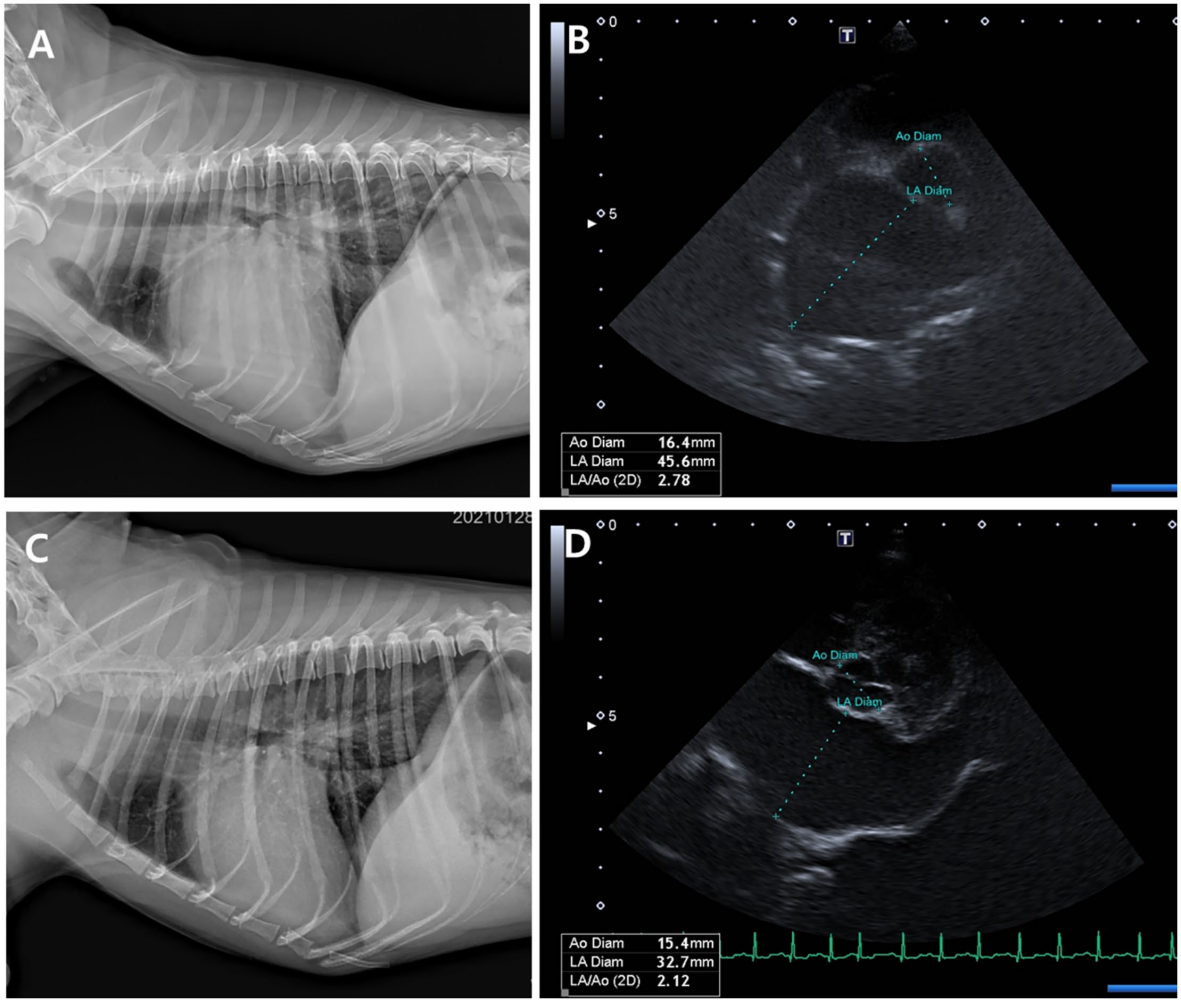
Thoracic radiographs **(A, C)** and echocardiographic images **(B, D)** from a Group 1 (test group) dog, taken before and 56 days after treatment with the combination tablet (ForteGold). Following treatment, the vertebral heart scale (VHS) and vertebral left atrial size (VLAS) on thoracic radiography decreased from 12.0 and 2.8 to 10.5 and 2.2, respectively **(A, C)**. Similarly, the left atrial-to-aortic root diameter ratio (LA/Ao) on echocardiography was reduced from 2.78 to 2.1 **(B, D)**.

Thoracic radiographs demonstrated a reduction in vertebral heart score (VHS) and pulmonary infiltration scores in both groups (Table 3; Figures 5, 6) However, the Group 1 (test group) exhibited faster resolution of pulmonary edema and cardiomegaly, with statistically significant improvement evident by Day 7 (*p* < 0.05), compared to Day 14 in the control group (Figures 5, 6).

### 3.5 Echocardiographic parameters

Both groups demonstrated reductions in LA/Ao, LVIDd/Ao, and MVE values over time (Tables 3, 4; Figures 5, 6). The magnitude of improvement was greater and occurred earlier in the Group 1 (test group). Significant reductions in LA/Ao ratio were observed as early as Day 14 in Group 1 (*p* < 0.05), compared to Day 28 in Group 2 (control; Figures 5, 6).

### 3.6 Compliance and administration

Owner-reported compliance was significantly higher in the Group 1 (test group) due to the convenience of fixed-dose tablet administration. Reports of missed doses or dosing errors were notably higher in the powdered medication group.

## 4 Discussion

In many parts of Asia, particularly in small animal veterinary clinics, it is common practice to manually combine and grind multiple cardiac medications into a single powdered formulation for ease of administration. While this method may seem convenient, it presents several significant pharmacological and clinical challenges (8–12). First, manual compounding often results in inaccurate dosing due to variable particle size, uneven distribution of active ingredients, and measurement errors. This can lead to subtherapeutic effects or drug toxicity, especially in patients requiring precise dosing over long-term management (11). Second, grinding tablets can compromise coating integrity and release profiles, especially in extended-release or enteric-coated formulations, which are designed for specific absorption characteristics. Mixing multiple drugs may also lead to unintended chemical interactions and reduced bioavailability (10). Third, powders often have poor palatability, leading to low owner compliance and frequent missed doses. This is a critical issue in chronic conditions like heart failure, where treatment adherence is essential for clinical stability (9). Fourth, improper mixing may cause uneven drug absorption, raising the risk of adverse effects such as electrolyte imbalances, renal stress, or gastrointestinal irritation, particularly when potent agents like diuretics or ACE inhibitors are involved (12). Last, unlike commercial formulations, compounded powders are often not subject to strict quality control, resulting in batch-to-batch variability and questionable stability (8). The test drug in this study was created to address the issues of compounded prescriptions.

Prior to the clinical trial, a bioequivalence test was conducted on the drugs used in the two groups (data not shown here). The results showed that most of the active ingredients in the medication administered in powder form had lower oral absorption and steady-state concentration compared to the same medication administered in tablet form. Furthermore, this study also demonstrates that the test drug, a fixed-dose combination tablet, is an effective and well-tolerated option for managing heart failure in dogs with MMVD, as similarly reported elsewhere (3). The clinical efficacy of this test drug was comparable or superior to traditional compounded medications, with faster symptom resolution and sustained improvements in both objective and subjective clinical parameters.

The improved outcomes observed in the test group may be attributed to enhanced dosing accuracy, superior drug bioavailability, and better owner compliance. These factors are critical in the long-term management of MMVD, where inconsistent medication delivery can lead to fluctuations in therapeutic response and increased risk of decompensation.

The observed reductions in NT-proBNP—a key prognostic biomarker for cardiac load—support the cardioprotective benefits of the fixed-dose formulation. Additionally, the stability of renal (SDMA, creatinine) and pancreatic (cPL) biomarkers reinforces the safety of the test drug, particularly in long-term use. In this study, the reduction in the cardiac biomarker NT-proBNP was more dramatic compared to the changes observed in diagnostic imaging studies. Further investigation is needed to determine whether this discrepancy is due to the cardiac biomarker responding more rapidly than imaging findings, or whether it may be attributed to statistical issues such as measurement variability or regression to the mean. Although NT-proBNP is a valuable biomarker for the detection and monitoring of cardiac disease in dogs, several factors may influence its accuracy and clinical interpretation. Measurement variability can arise from inter-assay and intra-assay differences, as well as inconsistencies in sample handling, storage, and timing. Additionally, biological noise—including the effects of age, body condition, renal function, and concurrent systemic illness—can obscure true changes in NT-proBNP concentration. It is also important to acknowledge the potential influence of non-biological factors such as the placebo effect and caregiver bias, particularly in clinical trials or subjective assessments of therapeutic response. Regression to the mean is another consideration; dogs with extreme baseline NT-proBNP values may demonstrate changes on subsequent testing that reflect statistical artifacts rather than genuine physiological improvement or deterioration. Furthermore, studies with small sample sizes are prone to overestimation or underestimation of NT-proBNP’s diagnostic or prognostic utility due to sampling variability and limited statistical power.

Radiographic and echocardiographic findings further substantiated clinical improvements, with Group 1 (test group) dogs showing earlier normalization of cardiac dimensions and pulmonary edema resolution. These objective findings align with previous studies emphasizing the importance of effective diuresis and neurohormonal blockade in MMVD management.

Serum SDMA and cPL are useful biomarkers in clinical practice; however, both are prone to high variability in dogs with congestive heart failure (CHF), as noticed in this study. For SDMA, fluctuations are often driven by transient changes in glomerular filtration rate (GFR), which may be influenced by decreased cardiac output, systemic hypotension, and aggressive diuresis—common features in this study population (13). These hemodynamic shifts can lead to reversible elevations in SDMA unrelated to intrinsic renal injury. Additionally, alterations in hydration status, such as dehydration or overhydration, can concentrate or dilute serum SDMA, further complicating interpretation. Similarly, cPL concentrations can be influenced by non-pancreatic factors in dogs with CHF (7). Reduced pancreatic perfusion, venous congestion, and systemic hypoxia can cause subclinical pancreatic stress or enzyme leakage, resulting in transient cPL elevations. Moreover, gastrointestinal disturbances (e.g., nausea, vomiting, inappetence) or systemic inflammation commonly associated with CHF may further modulate cPL levels, Aas noticed in this study population.

While compounded medications remain common in regions where commercial formulations are limited, this study highlights their drawbacks, particularly regarding dosing inconsistency and poor adherence. Fixed-dose tablets like this test drug may help overcome these challenges by simplifying treatment regimens and improving therapeutic reliability.

Dogs in ACVIM Stage C have developed signs of congestive heart failure (CHF) due to mitral valve disease (MVD), such as coughing, difficulty breathing, or exercise intolerance. Treatment focuses on managing symptoms, improving heart function, and improving quality of life. The main medications typically used are pimobendan, loop diuretics (either furosemide or torsemide), angiotensin-converting enzyme inhibitors (ACEi) and spironolactone (1). Because stage C refers to dogs that have developed clinical signs of heart failure as a result of mitral valve disease, or have had such signs in the past but are currently stable with treatment, there may be substantial variability in the severity of clinical signs among stage C dogs. Although there is some concern that a fixed dosing regimen may lead to overdosing in certain Stage C dogs, it also plays a preventive role by reducing the load on the left ventricle, thereby slowing the progression of cardiac enlargement and preventing deterioration into pulmonary edema. As a result, it may help delay the long-term progression of heart failure. Therefore, if no renal-related adverse effects occur, a slightly more aggressive pharmacologic approach is unlikely to be harmful to the patient. In fact, this study also confirmed that fixed dosing did not lead to any renal-related adverse effects during the study period, suggesting that this approach is not clinically problematic.

The use of fixed dosing regimens—particularly fixed-dose diuretics—in dogs with congestive heart failure (CHF) presents notable clinical challenges, especially in dogs at weight extremes (very small or very large breeds) or those with comorbid conditions such as chronic kidney disease (CKD). In very small dogs, fixed doses may result in relative overdosing, increasing the risk of excessive diuresis, dehydration, electrolyte disturbances (e.g., hypokalemia), and renal function deterioration. Conversely, in very large dogs, fixed dosing may lead to underdosing, contributing to persistent congestion, inadequate control of pulmonary edema, and delayed clinical improvement. However, since Sentorpil® ForteGold is formulated to allow dose adjustment through tablet splitting, it enables more precise and individualized dosing, thereby helping to mitigate these risks.

Although many of the components of the drugs used in this study could potentially affect patients’ systemic blood pressure, blood pressure changes were not monitored in this study. This is because blood pressure tends to vary significantly depending on the patient’s level of agitation at the time of examination rather than their actual cardiac condition, leading to substantial variability between measurements. Furthermore, most patients were not in critical conditions where continuous blood pressure monitoring would be essential. Therefore, no follow-up investigation on blood pressure was conducted.

This study has several limitations. First, although the sample size calculation is performed prior to study, the final cohort of 60 dogs remains relatively small to capture the full clinical heterogeneity of ACVIM stage C MMVD, particularly among dogs with comorbidities or of extreme body weights. Second, the 56-day follow-up period is relatively short for a chronic, progressive condition like MMVD, and the long-term durability of the observed clinical benefits remains unknown. Third, while the exclusion of blood pressure monitoring is explained, it should still be acknowledged as a limitation given that all tested drugs can influence systemic blood pressure and hemodynamic stability. Fourth, the manuscript refers to improved bioavailability of the fixed-dose tablet, yet no pharmacokinetic data are presented; this assumption should be framed as a hypothesis supported by clinical outcomes, pending confirmatory pharmacological studies. Fifth, although all dogs were categorized as ACVIM stage C, the study did not incorporate any internal stratification based on disease severity such as in the EPIC study (2). The absence of such stratification may limit the interpretation of treatment effects across varying degrees of cardiac remodeling or clinical decompensation within the same ACVIM stage. Lastly, although briefly mentioned, the potential impact of caregiver bias and placebo effect on subjective clinical assessments deserves stronger emphasis.

## 5 Conclusion

The test drug in this study offers a safe, effective, and convenient therapeutic option for managing MMVD-associated heart failure in dogs. Compared to compounded powder formulations, this test drug provides superior clinical outcomes, improved owner compliance, and better biomarker control without compromising safety. These findings support the integration of fixed-dose formulations into standard treatment protocols for canine MMVD. Further multicenter and long-term studies are warranted to validate these results and assess their impact on survival outcomes.

## Data Availability

The datasets presented in this study can be found in online repositories. The names of the repository/repositories and accession number(s) can be found in the article/supplementary material.
