# Correction: Clinical evaluation of a novel fixed-dose combination tablet (Sentorpil^®^ ForteGold) versus compounded powdered medications in dogs with myxomatous mitral valve disease: a randomized, double-blind study

**DOI:** 10.3389/fvets.2025.1687939

**Published:** 2025-09-11

**Authors:** Jiyoung Park, Jiyoon Lee, Sang-Joon Lee, Changbaig Hyun

**Affiliations:** Hyun Changbaig Animal Heart Institute, VIP Animal Medical Center, Seoul, Republic of Korea

**Keywords:** myxomatous mitral valve disease, fixed-dose combination tablet, canine cardiology, ForteGold, randomized clinical trial

The occurrence of “Sentorphil” has been updated to the correct spelling of “Sentorpil^®^” throughout the article.

The title of this article was erroneously given as: Clinical Evaluation of a Novel Fixed-Dose Combination Tablet (Sentorphil^®^ ForteGold) Versus Compounded Powdered Medications in Dogs with Myxomatous Mitral Valve Disease: A Randomized, Double-Blind Study.

The correct title of the article is: Clinical evaluation of a novel fixed-dose combination tablet (Sentorpil^®^ ForteGold) versus compounded powdered medications in dogs with myxomatous mitral valve disease: a randomized, double-blind study.

In **Abstract**, *Methods*, page 1.

The incorrect sentence previously stated:

“In a randomized, double-blind clinical trial, 60 client-owned dogs diagnosed with ACVIM stage C MMVD were assigned to receive either a novel fixed-dose combination tablet (Sentorphil^®^ ForteGold) or a compounded powdered mixture of torsemide, pimobendan, enalapril, and spironolactone.”

The corrected sentence appears below:

“In a randomized, double-blind clinical trial, 60 client-owned dogs diagnosed with ACVIM stage C MMVD were assigned to receive either a novel fixed-dose combination tablet (Sentorpil^®^ ForteGold) or a compounded powdered mixture of torsemide, pimobendan, enalapril, and spironolactone.”

In **Abstract**, *Discussion*, page 1.

The incorrect sentence previously stated:

“The fixed-dose combination tablet (Sentorphil^®^ ForteGold) offers a clinically effective and safer alternative to compounded powdered medications for managing MMVD in dogs.”

The corrected sentence appears below:

“The fixed-dose combination tablet (Sentorpil^®^ ForteGold) offers a clinically effective and safer alternative to compounded powdered medications for managing MMVD in dogs.”

In *Citation*, page 1:

The incorrect Citation previously stated:

“Park J, Lee J, Lee S-J and Hyun C (2025) Clinical evaluation of a novel fixed-dose combination tablet (Sentorphil^®^ ForteGold) versus compounded powdered medications in dogs with myxomatous mitral valve disease: a randomized, double-blind study. *Front. Vet. Sci*. 12:1622383. doi: 10.3389/fvets.2025.1622383.”

The corrected Citation appears below:

“Park J, Lee J, Lee S-J and Hyun C (2025) Clinical evaluation of a novel fixed-dose combination tablet (Sentorpil^®^ ForteGold) versus compounded powdered medications in dogs with myxomatous mitral valve disease: a randomized, double-blind study. *Front. Vet. Sci*. 12:1622383. doi: 10.3389/fvets.2025.1622383”

In **Introduction**, paragraph 4, page 2:

The incorrect sentence previously stated:

“This study aimed to evaluate the clinical efficacy, safety profile, and practical advantages of (Sentorphil^®^ ForteGold), a newly developed fixed-dose combination tablet, compared with traditional compounded powder formulations.”

The corrected sentence appears below:

“This study aimed to evaluate the clinical efficacy, safety profile, and practical advantages of (Sentorpil^®^ ForteGold), a newly developed fixed-dose combination tablet, compared with traditional compounded powder formulations.”

In **Materials and methods**, paragraph 1, page 2:

The incorrect sentence previously stated:

“Group 1 received (Sentorphil^®^ ForteGold) (Careside, Korea), a tablet containing torsemide (0.2 mg), pimobendan (0.6 mg), enalapril maleate (1.0 mg), and spironolactone (2.0 mg), dosed at one tablet per 2 kg of body weight, administered twice daily.”

The corrected sentence appears below:

“Group 1 received (Sentorpil^®^ ForteGold) (Careside, Korea), a tablet containing torsemide (0.2 mg), pimobendan (0.6 mg), enalapril maleate (1.0 mg), and spironolactone (2.0 mg), dosed at one tablet per 2 kg of body weight, administered twice daily.”

In **Discussion**, paragraph 10, page 10:

The incorrect sentence previously stated:

“However, since Sentorphil^®^ ForteGold is formulated to allow dose adjustment through tablet splitting, it enables more precise and individualized dosing, thereby helping to mitigate these risks.”

The corrected sentence appears below:

“However, since Sentorpil^®^ ForteGold is formulated to allow dose adjustment through tablet splitting, it enables more precise and individualized dosing, thereby helping to mitigate these risks.”

The original version of this article has been updated.

